# Refinement and partial validation of the UNESP-Botucatu multidimensional composite pain scale for assessing postoperative pain in horses

**DOI:** 10.1186/s12917-015-0395-8

**Published:** 2015-04-01

**Authors:** Marilda Onghero Taffarel, Stelio Pacca Loureiro Luna, Flavia Augusta de Oliveira, Guilherme Schiess Cardoso, Juliana de Moura Alonso, Jose Carlos Pantoja, Juliana Tabarelli Brondani, Emma Love, Polly Taylor, Kate White, Joanna C Murrell

**Affiliations:** Veterinary Medicine Department, Universidade Estadual de Maringá, Estrada da Paca s/n, Umuarama, Brazil; Department of Veterinary Surgery and Anesthesiology, College of Veterinary Medicine and Animal Science, UNESP – Univ Estadual Paulista, Botucatu, SP 18618970 Brazil; School of Veterinary Science, Langford House, Langford, UK; Taylor Monroe, Ely, Cambridgeshire UK; School of Veterinary Medicine and Science, University of Nottingham, Nottingham, UK

**Keywords:** Validity, Reliability, Responsiveness, Specificity, Sensitivity, Relevance, Horse, Pain

## Abstract

**Background:**

Quantification of pain plays a vital role in the diagnosis and management of pain in animals. In order to refine and validate an acute pain scale for horses a prospective, randomized, blinded study was conducted. Twenty-four client owned adult horses were recruited and allocated to one of four following groups: anaesthesia only (GA); pre-emptive analgesia and anaesthesia (GAA,); anaesthesia, castration and postoperative analgesia (GC); or pre-emptive analgesia, anaesthesia and castration (GCA). One investigator, unaware of the treatment group, assessed all horses at time-points before and after intervention and completed the pain scale. Videos were also obtained at these time-points and were evaluated by a further four blinded evaluators who also completed the scale. The data were used to investigate the relevance, specificity, criterion validity and inter- and intra-observer reliability of each item on the pain scale, and to evaluate construct validity and responsiveness of the scale.

**Results:**

Construct validity was demonstrated by the observed differences in scores between the groups, four hours after anaesthetic recovery and before administration of systemic analgesia in the GC group. Inter- and intra-observer reliability for the items was only satisfactory. Subsequently the pain scale was refined, based on results for relevance, specificity and total item correlation.

**Conclusions:**

Scale refinement and exclusion of items that did not meet predefined requirements generated a selection of relevant pain behaviours in horses. After further validation for reliability, these may be used to evaluate pain under clinical and experimental conditions.

## Background

Recognition of pain-related behaviours in animals is difficult due to inter-species and individual variation [1], yet it is universally acknowledged that improvements in pain assessment may facilitate diagnosis and analgesic treatment in horses. Previous studies have developed scales to assess equine orthopaedic [2] and abdominal pain [3-5]. However, to our knowledge, there are no published studies investigating pain scales in horses undergoing soft tissue surgery or experiencing pain of a similar intensity to that associated with castration.

There are established psychometric methods for developing and refining structured questionnaires of abstract constructs such as acute pain in humans. This approach can be adopted for similar purposes in animals. Initially the items to be assessed must be collected and refined for inclusion in the questionnaire. Thereafter the scale must be scrutinized for both content and face validity and finally the scale must undergo reliability testing [6]. Furthermore the instrument should be responsive and be able to measure changes as a result of an intervention such as a painful event, or analgesic administration [2,7].

The aim of this study was to refine and validate a new acute pain scale for the assessment of mild or moderate pain in horses, and to evaluate its reliability.

## Results

The GA group included four geldings and two mares (mean ± SD, 332 ± 48 kg and 9 ± 3 years old); the GAA group included three geldings and three mares (369 ± 68 kg and 10 ± 5 years old); the GC group comprised of six male horses (319 ± 48 kg and 4 ± 2 years old) and GCA also included six male horses (302 ± 27 kg and 4 ± 2 years old). Surgery and anaesthesia lasted approximately 45 minutes in all cases. Complete data were obtained from twenty horses. Four horses had missing data points; one horse (GA) at T4 and T6 and one horse (GCA) at T24 due to abdominal discomfort, which recovered after clinical treatment, one horse (GAA) at T24, due to technical problems with the camera and one horse (GC) at T24, due to postoperative haemorrhage.

**Figure 1:**
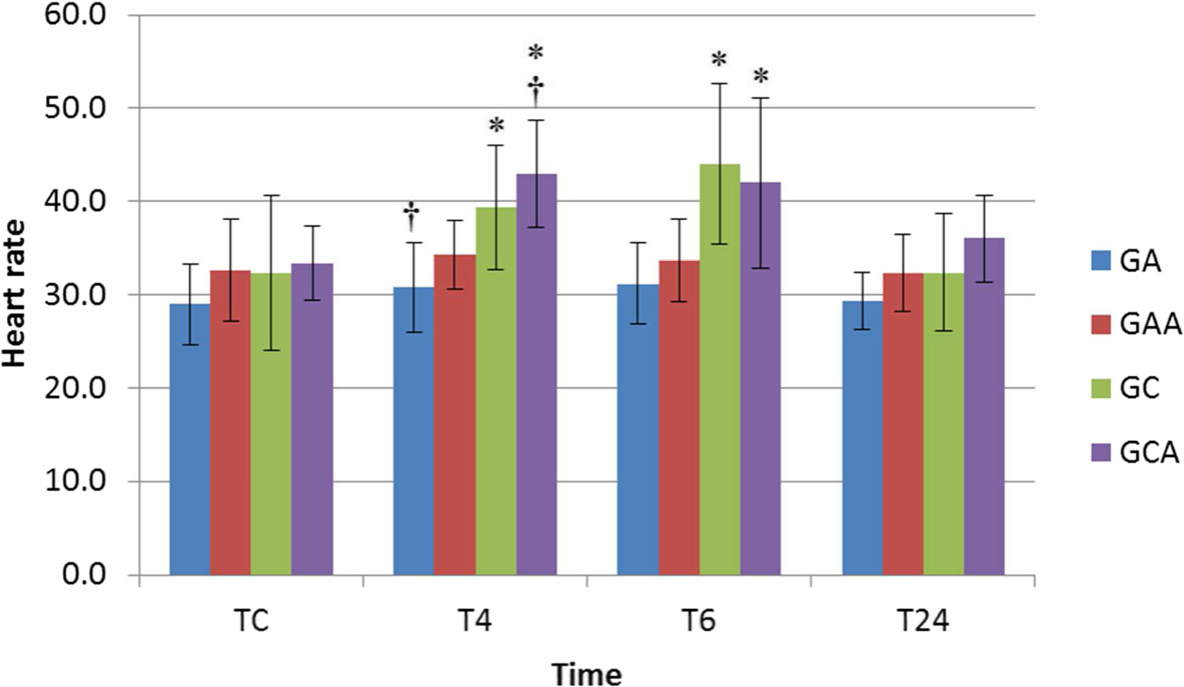
**Heart rate before and after anaesthesia (GA), analgesia and anaesthesia (GAA), analgesia, anaesthesia and castration (GCA) and anaesthesia, castration and analgesia (GC) in horses.** *Statistically significant difference between assessment time points within each group. † Significant difference between GA and GCA.

Content validity of the items included in the scale are shown in Table 1. The score for each item, the relevance, specificity and item-total correlation are shown in Table 2. A refined pain scale was produced after exclusion of the categories that did not show at least one item with adequate relevance and specificity. Heart rate was the only physiological variable retained in the pain scale, as it was the only one that differed over time (Figure 1). Comparison of the total scores between groups and at the different assessment time points was performed to confirm construct validity. At T4, pain scores were greater in GC than in the other groups, and greater in GCA than in GAA. Even after the administration of analgesics at T6, GC scores were still greater than GA and GAA, and GCA scores were greater than in GAA. At the 24-hour time point (T24) the scores of GC and GCA were still greater than those horses in GAA. There were no differences with time in scores for GA and GAA. In GC the scores at T4 were greater than at T6 and both were greater than TC (prior to anaesthesia and or surgery) and T24. The scores of GCA were greater in T4 than TC and T24.

**Table 1:** **Variables, criteria, scores, content validity and reproducibility of the acute pain scale in horses**

**Variable**		**Criteria**	**Score**	**Content validity** ^**1**^	**Reproducibility** ^**2**^					
					**EV1 X EV2**	**EV1 X EV3**	**EV1 X EV4**	**EV2 X EV3**	**EV2 X EV4**	**EV3 X EV4**
Posture	Positioning in the stall	The horse’s head is at the outside door	0	0.7	0.6 (0.4-0.7)	0.6 (0.4-0.8)	0.6 (0.5-0.8)	0.7 (0.5-0.8)	0.5 (0.3-0.7)	0.6 (0.4-0.7)
		The horse is inside the stall, but looking at the outside door. Observing the environment	1	*	0.5 (0.3-0.7)	0.4 (0.2-0.6)	0.5 (0.3-0.7)	0.5 (0.3-0.7)	0.3 (0.1-0.6)	0.4 (0.2-0.6)
		The horse is eating	0	*	0.7 (0.4-1.0)	0.7 (0.5-1.0)	0.6 (0.3-0.9)	0.8 (0.6-1.0)	0.7 (0.4-1.0)	0.7 (0.4-1.0)
		The horse is not close to the outside stall door and does not look interested in the environment	2	1	0.9 (0.6-1.0)	0.6 (0.3-1.0)	0.7 (0.4-1.0)	0.7 (0.4-1.0)	0.8 (0.6-1.0)	0.8 (0.6-1.0)
	Head position	Above the withers or grazing	0	1	0.4 (0.1-0.7)	0.4 (0.1-0. 7)	0.5 (0.2-0.7)	0.6 (0.3-1.0)	0.6 (0.2-0.9)	0.8 (0.6-1.0)
		At the level of the withers	1	*	0.3 (0–0.7)	0.3 (0–0.6)	0.3 (0–0.6)	0.5 (0.1-1.0)	0.3 (−0.2-0.7)	0.5 (−0.1-1.0)
		Below the withers but not eating	2	1	NE	NE	NE	1.0 (1.0-1.0)	0.7 (0.04-1.0)	0.7 (0.04-1.0)
Interactive behaviour	Response to opening the door	The horse moves towards the door or is already positioned at the outside door	0	0.7	0.7 (0.6-0.9)	0.7 (0.5-0.9)	0.4 (0.2-0.6)	0.7 (0.6-0.9)	0.4 (0.2-0.6)	0.5 (0.3-0.8)
		The horse looks at the door, but does not move towards the door	1	0.7	0.6 (0.4-0.8)	0.5 (0.2-0.7)	0.3 (0.1-0.6)	0.6 (0.4-0.8)	0.3 (0.1-0.6)	0.5 (0.2-0.7)
		The horse does not respond to opening the door	2	0.7	0.4 (0–0.8)	0 (0–0)	0.5 (0.0-0.9)	0.3 (−0.2-0.8)	0.2 (−0.2-0.7)	0 (0–0)
	Response to approach and presence of the observer	Moves towards or looks to the observer	0	0.7	0.2 (0.1-0.3)	0.3 (0.1-0.4)	0 (0–0.1)	0.5 (0.3-0.7)	0 (−0.1-0.2)	0.1 (−0.1-0.3)
		Moves away from the observer	1	0.7	0.1 (−0.2-0.4)	0.2 (−0.2-0.6)	0 (0–0)	0 (0–0)	0 (0–0)	0 (0–0)
		Does not move	2	0.7	0.2 (0–0.3)	0.2 (0–0.4)	0.1 (0–0.1)	0.5 (0.2-0.7)	0.1 (−0.1-0.3)	0.1 (0–0.4)
Appetite	Appetite for hay	The horse eats hay	0	0.7	0.2 (−0.2-0.6)	0.7 (0–1.0)	0.7 (0–1.0)	0.3 (−0.1-0.7)	0.3 (−0.1-0.7)	1.0 (1.0-1.0)
		The horse does not eat hay	1	0.7	0.3 (−0.2-0.7)	0.7 (0–1.0)	0.7 (0–1.0)	0.3 (−0.1-0.8)	0.3 (−0.1-0.8)	1.0 (1.0-1.0)
	Response to concentrate food	Moves to the food and eats	0	0.7	−0.2 (−0.3- -0.1)	0.4 (0–0.7)	0.3 (0–0.6)	0.2 (0.1-0.5)	−0.1 (−0.2- -0.1)	0.4 (0–0.7)
		Hesitates to move towards the food, but eats	1	0.7	−0.1 (−0.2- -0.1)	0.6 (0.2-0.9)	0.4 (0.1-0.8)	0.2 (−0.1-0.5)	−0.1 (−0.2- -0.1)	0.4 (0–0.7)
		Shows no interest in food, not eating	2	0.7	NE	NE	NE	NE	NE	NE
Activity	Locomotion	The horse moves freely alone	0	0.7	0.1 (0–0.2)	0.5 (0.3-0.8)	0.3 (0.1-0.6)	0.1 (0–0.2)	0.1 (0–0.2)	0.3 (0.1-0.6)
		The horse does not move, or is reluctant to move	1	1	0.1 (0–0.3)	0.5 (0.3-0.8)	0.5 (0.2-0.8)	0.1 (0–0.2)	0.1 (0–0.3)	0.5 (0.2-0.9)
		The horse is agitated, restless	2	1	NE	NE	NE	NE	0.1 (−0.2-0.5)	NE
	Locomotion when led by the evaluator	The horse moves freely when led	0	0.7	0 (−0.2-0.1)	0.3 (0–0.7)	0.7 (0.4-1.0)	0.1 (0–0.3)	0.1 (0–0.2)	0.6 (0.3-1.0)
		The horse does not move, or is reluctant to move when led	1	1	0.1 (0–0.2)	0.5 (0.1-0.9)	0.8 (0.5-1.0)	0.1 (0–0.3)	0.1 (0–0.2)	0.6 (0.3-1.0)
		The horse is agitated, restless	2	1	NE	NE	NE	NE	NE	NE
**Variable**		**Criteria**	**Score**	**Content validity** ^**1**^	**Reproducibility** ^**2**^					
					**EV1 X EV2**	**EV1 X EV3**	**EV1 X EV4**	**EV2 X EV3**	**EV2 X EV4**	**EV3 X EV4**
Palpation	Response to palpation of the painful area (approximately 3 cm besides the wound)	No response or change in relation to pre-procedure palpation response of the surgical wound	0	1	0.5 (0.3-0.6)	0.7 (0.5-0.8)	0.7 (0.5-0.8)	0.7 (0.5-0.8)	0.6 (0.5-0.3)	0.7 (0.6-0.9)
		Mild reaction to palpation of the surgical wound	1	1	0.3 (0.1-0.5)	0.5 (0.3-0.7)	0.6 (0.5-0.8)	0.4 (0.3-0.6)	0.3 (0.1-0.5)	0.4 (0.2-0.6)
		Violent reaction to palpation of the surgical wound	2	1	0.3 (0–0.6)	0.4 (0.2-0.7)	0.6 (0.3-0.9)	0.6 (0.4-0.8)	0.4 (0.1-0.7)	0.5 (0.2-0.7)
Interactive behaviour	Response to an auditory stimulus (clap hands)	Moves and/or pays attention with ears or head movements	0	*	0.3 (0.1-0.5)	0.4 (0.1-0.6)	0.3 (0–0.5)	0.4 (0.1-0.6)	0.5 (0.2-0.7)	0.6 (0.4-0.8)
		Calm, no response	1	*	0.4 (0.2-0.7)	0.5 (0.-3-0.8)	0.4 (0.1-0.6)	0.4 (0.1-0.6)	0.5 (0.2-0.7)	0.6 (0.4-0.8)
		No response to auditory stimulus due to prostration	2	*	NE	NE	NE	NE	NE	NE
Miscellaneous behaviours	Looking at the flank	The horse does not look at the flank	0	0.7	0.4 (0.2-0.7)	0.3 (0.1-0.5)	0.4 (0.2-0.7)	0.5 (0.3-0.7)	0.5 (0.3-0.7)	0.6 (0.4-0.7)
		The horse looks at the flank	1	0.7	0.4 (0.2-0.7)	0.3 (0.1-0.5)	0.4 (0.2-0.7)	0.5 (0.3-0.7)	0.5 (0.3-0.7)	0.6 (0.4-0.8)
	Kicking at the abdomen	The horse does not kick the abdomen	0	0.7	0 (0–0)	0.1 (−0.1-0.4)	0.3 (0–0.7)	0 (−0.1-0)	0 (0–0)	0.3 (0–0.6)
		The horse kicks at the abdomen	1	0.7	NE	0.2 (0–0.5)	0.5 (0–0.9)	NE	NE	0.3 (0–0.6)
	Lifting hind limbs	No lifting of hind limbs	0	*	0.5 (0.3-0.7)	0.5 (0.4-0.7)	0.6 (0.4-0.8)	0.4 (0.2-0.6)	0.5 (0.3-0.7)	0.6 (0.4-0.8)
		Lifting hind limbs	1	*	0.3 (0.1-0.5)	0.4 (0.2-0.5)	0.5 (0.3-0.7)	0.2 (0–0.4)	0.3 (0.1-0.5)	0.6 (0.4-0.8)
		Lifting hind limbs and extending the head	2	*	0.2 (0–0.5)	0 (0–0)	0 (0–0)	0 (0–0)	0.1 (−0.1-0.3)	0 (0–0)
	Head movement	Head straight ahead most of the time	0	*	0.1 (0–0.2)	0.1 (−0.2-0.4)	0.6 (0.3-0.9)	0 (−0.1-0.2)	0.2 (0–0.4)	0.4 (0–0.7)
		Lateral and/or vertical occasional head movements	1	*	0 (−0.1-0.1)	0 (−0.1-0)	0.5 (0.2-0.8)	0 (−0.1-0.2)	0.1 (0–0.3)	0.3 (0–0.7)
		Lateral and/or vertical continuous head movements	2	*	0 (0–0)	NE	NE	NE	NE	NE
	Pawing on the floor (fore limbs)	Quietly standing, no pawing	0	0.7	0.2 (0–0.5)	0.2 (0–0.4)	0.2 (0–0.5)	0.3 (0–0.5)	0.5 (0.2-0.7)	0.7 (0.5-0.9)
		Pawing	1	1	0.3 (0–0.6)	0.2 (0–0.4)	0.2 (0–0.5)	0.4 (0.1-0.7)	0.5 (0.2-0.7)	0.7 (0.5-0.9)
	Others	Moving the tail sharply and repeatedly	1	*	0.1 (−0.1-0.2)	0.3 (0.1-0.4)	0.1 (−0.1-0.4)	0.2 (0–0.4)	0.2 (0–0.4)	0.4 (0.3-0.6)
		Moving the tail sharply and repeatedly and lifting the hind limbs	2	*	0.4 (0.1-0.7)	0.4 (0.2-0.6)	0.4 (0.1-0.7)	0.3 (0.1-0.5)	0.5 (0.2-0.8)	0.5 (0.3-0.7)
		Partial penis protrusion	1	*	0.4 (0.1-0.5)	0 (−0.2-0.2)	0.1 (−0.1-0.4)	0.2 (0–0.5)	0.1 (−0.1-0.3)	0.3 (0–0.5)
		Penis protrusion	0	*	0.4 (0.2-0.6)	0.2 (0–0.4)	0.5 (0.2-0.7)	0.4 (0.1-0.7)	0.5 (0.3-0.8)	0.7 (0.5-0.9)

^1^Content validity obtained by the arithmetic mean of the scores given by the three evaluators for each item of the scale [20].

^2^Inter-observer reproducibility was tested with the Kappa coefficient comparing video analysis among observers. > 0.7 - Excellent, 0.4 to 0.7 - moderate; <0.4 - poor reliability [21].

EV – Evaluator. NE – not evaluated as there were not sufficient data for statistical analysis (the behaviour was either very infrequent or not observed). * Item included after content validation.

**Table 2:** **Relevance, specificity and item-total correlation of each item and categories of the scale in horses submitted to castration or only anaesthesia**

**Variable**	**Criteria**	**Specificity (%)** ^**1**^	**Relevance (confidence interval)** ^**2**^			**Item-total correlation** ^**3**^
			**GA x GC**	**GAA X GC**	**GCA x GC**	
**Positioning in the stall**	**The horse’s head is at the outside door**	**78.3**	18.6 (4.4-78.9)*	48.3 (10.0-233.1)*	**0.1 (0–0.4)**	**0.4**
	**The horse is inside the stall, but looking at the outside door, observing the environment**	**19.5**	**0.6 (0.2-2.0)**	**0.1 (0.0-0.7)**	**1.9 (0.6-6.16)**	
	**The horse is eating**	**0.9**	**NE**	**NE**	**NE**	
	**The horse is not close to the outside stall door and does not look interested in the environment**	**0.9**	**NE**	**NE**	**NE**	
Head position	Above the withers or grazing	95.0	NE	NE	NE	0.2
	At the level of the withers	3.3	NE	NE	NE	
	Below the withers but not eating	0.0	NE	NE	NE	
Response to opening the door	The horse moves towards the door or is already positioned at the outside door	15.8	1.0 (0.2-3.7)	1.5 (0.4-6.0)	1.9 (0.6-6.5)	0.3
	The horse looks at the door, but does not move towards the door	15.8	0.7 (0.2-3.3)	0.3 (0–1.7)	0.5 (0.1-1.7)	
	The horse does not respond to opening the door	1.7	NE	NE	NE	
Response to approach and presence of the observer	Moves towards or looks to the observer	84.7	0.3 (0.1-1.5)	0.2 (0.1-0.9)	1.4 (0.3-6.1)	0.2
	Moves away from the observer	2.5	NE	NE	NE	
	Does not move	12.7	3.8 (0.8-18.8)	4.6 (1.0-21.2)*	0.7 (0.1-3.7)	
Appetite for hay	The horse eats hay	99.1	NE	NE	NE	0.2
	The horse does not eat hay	0.8	NE	NE	NE	
Response to concentrate food	Moves to the food and eats	90.7	NE	NE	NE	0.2
	Hesitates to move towards the food, but eats	8.0	NE	NE	NE	
	Shows no interest in food, not eating	0	NE	NE	NE	
**Locomotion**	**The horse moves freely alone**	**84.2**	47.6 (4.9-464.0)*	5.8 (1.6-21.3)*	**0.2 (0–0.6)**	**0.4**
	**The horse does not move, or is reluctant to move**	**10.0**	**0 (0–0.2)**	**0.2 (0.1-0.7)**	6.2 (1.6-23.2)*	
	**The horse is agitated, restless**	**5.8**	**NE**	**NE**	**NE**	
**Locomotion when led by the evaluator**	**The horse moves freely when led**	**86.5**	108.2 (4.4- > 999.9)*	68.1 (4.9-946.5)*	**0 (<0.001-0.1)**	**0.4**
	**The horse does not move, or is reluctant to move when led**	**12.2**	**NE**	**NE**	**NE**	
	**The horse is agitated, restless**	**0.0**	**NE**	**NE**	**NE**	
**Variable**	**Criteria**	**Specificity (%)** ^**1**^	**Relevance (confidence interval)** ^**2**^			**Item-total correlation** ^**3**^
			**GA x GC**	**GAA X GC**	**GCA x GC**	
**Response to palpation of the painful area (approximately 3 cm besides the wound)**	**No response or change in relation to pre-procedure palpation response of the surgical wound**	**57.1**	4.1 (1.1-15.)*	18.8 (4.6-76.0)*	0.3 (0.1-1.1)*	**0.4**
	**Mild reaction to palpation of the surgical wound**	**29.4**	**0.1 (0–0.5)**	**0.1 (0–0.5)**	1.9 (0.6-5.9)*	
	**Violent reaction to palpation of the surgical wound**	**13.4**	2.1 (0.6-7.8)*	0.1 (0–1.3)*	1.7 (0.4-7.5)*	
Response to an auditory stimulus (clap hands)	Moves and/or pays attention with ears or head movements	80.0	6.8 (0.5-84.0)	4.9 (0.7-33.6)	0.5 (0.1-2.0)	0.3
	Calm, no response	18.6	NE	NE	NE	
	No response to auditory stimulus due to prostration	0.0	NE	NE	NE	
**Looking at the flank**	**The horse does not look at the flank**	**84.6**	46.7 (9.9-219.0)*	128.9 (21.2-782.7)*	0.1 (0–0.3)*	**0.4**
	**The horse looks at the flank**	**15.4**	**0 (0–0.1)**	**0 (0–0)**	11.7 (3.1-43.7)*	
**Kicking at the abdomen**	**The horse does not kick the abdomen**	**95.8**	**NE**	**NE**	**NE**	**0.3**
	**The horse kicks at the abdomen**	**2.5**	**NE**	**NE**	**NE**	
**Lifting hind limbs**	**No lifting of hind limbs**	**74.6**	18.0 (3.9-81.9)*	96.5 (16.4-566.4)*	**0.1 (0–0.4)**	**0.6**
	**Lifting hind limbs**	**22.0**	**0.2 (0.1-0.8)**	**0 (0–0.2)**	4.6 (1.3-16.1)*	
	**Lifting one of the hind limbs and extending the head**	**3.4**	**NE**	**NE**	**NE**	
**Head movement**	**Head straight ahead most of the time**	**87.1**	91.2 (10.1-822.7)*	32.4 (6.7-155.7)*	**0.1 (0–0.3)**	**0.4**
	**Lateral and/or vertical occasional head movements**	**12.1**	**NE**	**NE**	**NE**	
	**Lateral and/or vertical continuous head movements**	**0.9**	**NE**	**NE**	**NE**	
**Pawing on the floor (fore limbs)**	**Quietly standing, no pawing**	**89.7**	17.5 (3.6-84.9)*	38.9 (6.6-230.1)*	**0.1 (0–0.5)**	**0.4**
	**Pawing**	**9.4**	**0.1 (0–0.4)**	**0 (0–0.2)**	4.1 (1.12-14.1)*	
Others	Moving the tail sharply and repeatedly	21.7	0.9 (0.2-3.5)	0.5 (0.1-2.1)	1.8 (0.5-6.7)	0.5
	Moving the tail sharply and repeatedly and lifting the hind limbs	9.2	0 (0–0.2)	0 (0–0.2)	2.4 (0.9-6.9)	
	Partial penis protrusion	23.2	2.3 (0.4-12.5)	8.5 (1.6-4.2)	0.4 (0–2.0)	0.2
	Penis protrusion	47.4	NE	NE	NE	
			**GA x GC**	**GAA X GC**	**GCA x GC**	
**Heart rate**		**-**	**-**	**-**	**-**	**0.6**
Respiratory rate		-	-	-	-	0.5
Systolic blood pressure		-	-	-	-	0.4
Digestive sounds		-	-	-	-	0.3

The categories written in bold letters were used for the sum of the total score. NE – not evaluated as there were not sufficient data for statistical analysis (the behaviour was either infrequently or not observed). GA – Anaesthesia only (n = 6). GAA – Pre-emptive analgesia followed by anaesthesia (n = 6). GC – Anaesthesia, castration and postoperative analgesia administered four hours after surgery (n = 6). GCA – Pre-emptive analgesia, followed by anaesthesia and castration (n = 6).

^1^The specificity of each item was evaluated by investigating if that particular behaviour was present or not at TC, moment without pain, in all observations from all evaluators, in all animals from all groups. Specificity was classified as excellent (0–4.9%), good (5–14.9%), moderate (15–29.9%), or nonspecific (≥30%).

^2^Relevance was tested based on the possibility of distinguishing the behaviour in T4 in GC compared to the other groups. The item was considered relevant when that item differentiated GC from the other three groups, or when GC was different from animals without pain (GA and GAA). The item was considered irrelevant when there were no differences between GC and the other groups. Asterisks (*) indicate differences between groups.

^3^Criterion validity for item-total correlation with Pearson correlation test (weak < 0.30; moderate 0.31 – 0.60; strong 0.61 – 0.9; very strong 0.91 -1.0) [8].

The percentage increase in pain score in GC between TC and T4 was 282%, and the scores decreased by 39% and 61% of T4 at T6 and T24 respectively (Table 3).

**Table 3:** **Median (minimal and maximal value) pain scores in horses undergoing castration or anaesthesia only**

**Treatment**	**GA**	**GAA**	**GC**	**GCA**
**Moment**				
TC	3 (0–11)	2 (0–11)	4 (0–11)^C^	4 (0–10)^B^
T4	5 (1–14)^bc^	5 (2–14)^c^	14 (7–25)^aA^	11 (3–16)^bA^
T6	4 (1–6)^bc^	4 (1–7)^c^	10.5 (5–16)^aB^	6.5 (1–19)^abAB^
T24	3 (0–8)^ab^	2 (0–7)^b^	8 (1–14)^aC^	6.5 (2–13)^aB^

GA – Anaesthesia only (n = 6). GAA – Pre-emptive analgesia followed by anaesthesia (n = 6). GC – Anaesthesia, castration and postoperative analgesia administered four hours after surgery (n = 6). GCA – Pre-emptive analgesia, followed by anaesthesia and castration (n = 6). TC - before surgery and/or anaesthesia, T4 - maximum score of pain until 4 hours after anaesthetic recovery, T6 - six hours after anaesthetic recovery, T24 - 24 hours after anaesthetic recovery.

Different small letters indicate differences between groups (rows – a > b > c); different capital letters indicate differences between time points in the same group (columns – A > B > C).

Results of the criterion validation of the scores assigned to each item of the scale (derived by comparing the different evaluators’ scores to the standard evaluator’s), showed moderate to excellent variability for “positioning in the stall”, “appetite for hay” and “response to palpation of the groin”. With the exception of one evaluator, reliability for the item “locomotion” ranged from moderate to excellent. The horses’ response to opening the door and head movements showed moderate variability. “Appetite for concentrate/pelleted feed,” “looking at the flank”, “raising the hind limbs” and “tail movements” showed poor to moderate variability. Variability was also poor for the remaining items or otherwise the number of observations was low and therefore it was not possible to perform statistical analysis.

**Figure 2:**
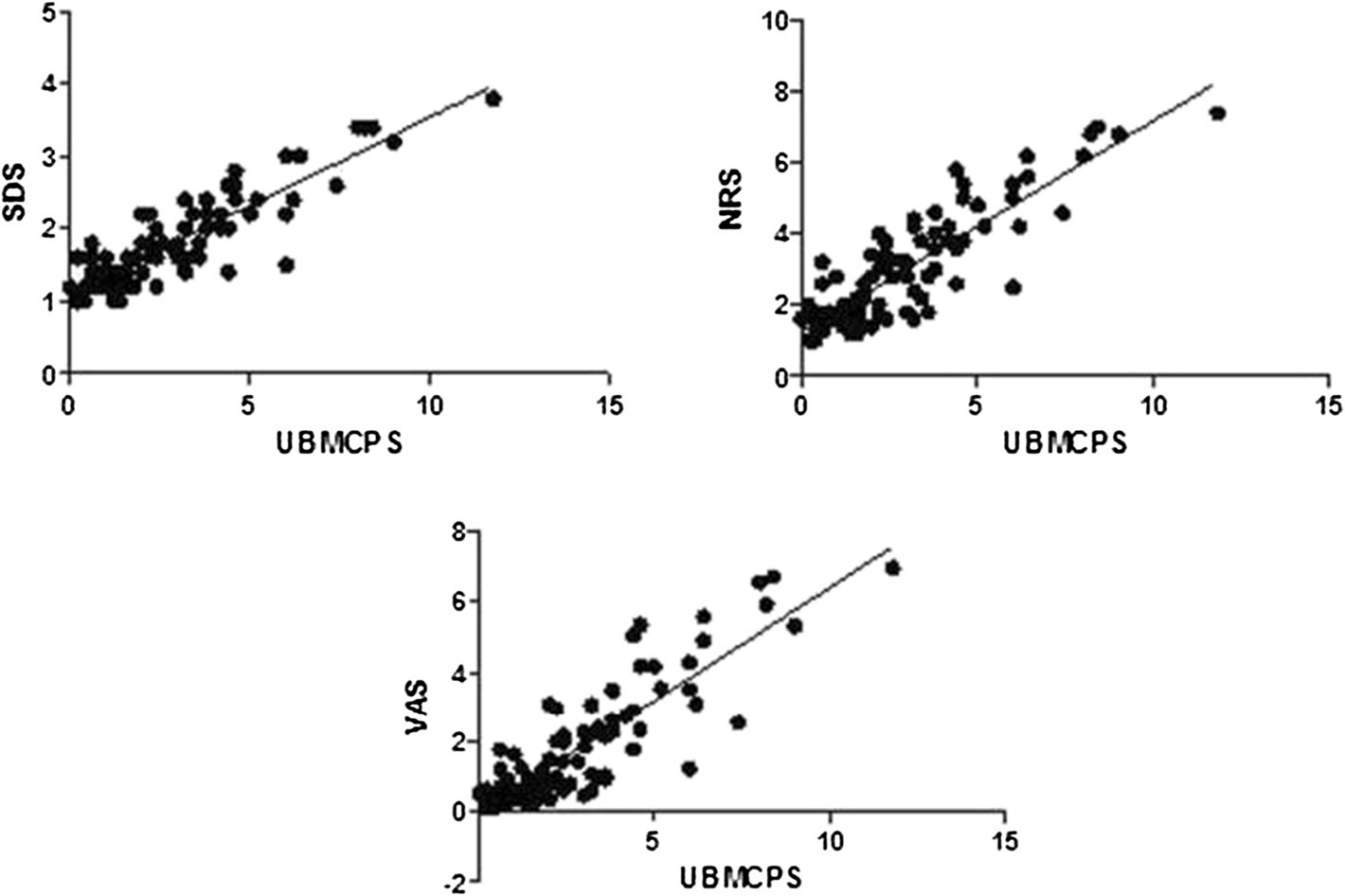
**Pearson correlation between the UNESP-Botucatu Multidimensional Composite Pain Scale (UBMCPS) and the Simple Descriptive Scale (SDS – r = 0.88, P < 0.0001).** Numerical Rate Scale (NRS – r = 0.87, P < 0.0001) and Visual Analogical Scale (VAS – r = 0.86, P < 0.0001).

Results of the criterion validation investigated by item-total correlation are presented in Table 2. The convergent validity was confirmed by positive correlation between the refined scale and the numerical (0.87), visual analogue (0.86) and simple descriptive scales (0.88), (see Figure 2) which were also assessed [8].

The reproducibility of each item, defined by the ability to obtain the same results in repeated assessments by different evaluators [9], was evaluated by measurement of inter-observer reliability and data are shown in Table 1.

The repeatability of each item, investigated by intra-observer reliability, was moderate to excellent for “positioning in the stall” and “kicking at the abdomen”, but ranged from poor to moderate for interactive behaviour, “lifting of hind limbs” and “penis protrusion”. It was also moderate for “locomotion when the horse was led by the evaluator”, “response to palpation” of the painful area (groin), “response to an auditory stimulus”, “pawing at the floor” and “moving the tail”.

There was no difference between groups in physiological parameters, except at time point T4 when heart rate was lower in GA than in GCA (P = 0.04). Heart rate was higher in GC and GCA at T4 and T6 compared to the other assessment time points. Item-total correlation was moderate for all physiological data.

After the refinement of the data based on the specificity, relevance and criterion validity, a modified acute pain scale was tested (Table 4).

**Table 4:** **Refined acute pain scale in horses submitted to castration after the refinement of the data based on the specificity, relevance and criterion validity**

**Variable**	**Criteria**	**Score**
Positioning in the stall	The horse’s head is at the outside door	0
	The horse is inside the stall, but looking at the outside door, observing the environment	1
	The horse is eating	0
	The horse is not close to the outside stall door and does not look interested in the environment	2
Locomotion	The horse moves freely alone	0
	The horse does not move, or is reluctant to move	1
	The horse is agitated, restless	2
Locomotion when led by the evaluator	The horse moves freely when led	0
	The horse does not move, or is reluctant to move when led	1
	The horse is agitated, restless	2
Response to palpation of the painful area (approximately 3 cm besides the wound)	No response or change in relation to pre-procedure palpation response of the surgical wound	0
	Mild reaction to palpation of the surgical wound	1
	Violent reaction to palpation of the surgical wound	2
Looking at the flank	The horse does not look at the flank	0
	The horse looks at the flank	1
Kicking at the abdomen	The horse does not kick the abdomen	0
	The horse kicks at the abdomen	1
Lifting hind limbs	No lifting of hind limbs	0
	Lifting hind limbs	1
	Lifting hind limbs and extending the head	2
Head movement	Head straight ahead most of the time	0
	Lateral and/or vertical occasional head movements	1
	Lateral and/or vertical continuous head movements	2
Pawing on the floor (fore limbs)	Quietly standing, no pawing	0
	Pawing	1
Heart rate (compared to initial values)	25-50% increase	1
	>50% increase	2

## Discussion

The pain scale demonstrated construct and content validity; however intra- and inter-observer reliability for the items were only satisfactory, suggesting that refinement and readjustment of the items was required.

Construct validity was demonstrated by the observed differences in scores between the groups at T4 [10]. The differences between GC, GA and GAA show that the scale is able to differentiate between horses with and without pain. Furthermore, in view of the fact that pain scores were different between GC and GCA, the scale was also able to identify different pain intensities. The ability of the scale to measure pain was confirmed by its responsiveness, seen in the change in GC scores between T4, T6 and T24 [2], and by the percentage change in pain scores after surgery and in response to analgesic administration [11].

In both groups that did not undergo surgery (GA, GAA) male and female horses were included, which may have been a limitation of the study; since the scale was based on assessment of behaviour, sex differences in behaviour may produce differences in scores between groups at TC. However, this did not occur and scores in the different sexes were similar. The absence of a difference between groups before surgery and/or anaesthesia provides an alternative means to confirm construct validity of the pain scale. Response to stress may mimic pain behaviour, and since it might be predicted that stallions would be more agitated prior to surgery, the lack of a difference between treatment groups in baseline scores shows that the construct validity of the scale was not compromised [6].

Another aspect that may have confused the interpretation of pain behaviours, especially in relation to assessments requiring the evaluator to interact with the horse, was the time allowed for each horse to acclimatise to the stable, and to the investigator who interacted with the horses. Variability in the degree of this initial interaction between each horse and the investigator was expected. However, in order to limit this, one of the inclusion criteria for the study was that the horse must be halter trained and used to interacting with people.

Another limitation of the study is that the pain model used here (castration) probably results in only mild to moderate pain. Therefore the scale should also be tested under conditions considered to cause more severe pain and be validated under these circumstances as well.

To date there are no validated pain scales that measure mild to moderate soft tissue pain in horses and so criterion validity was evaluated by the contrast in variability between the standard evaluator and the other evaluators [7,11]. The total scores from the pain scale were correlated with three other classical scales used to measure clinical pain in animals and a strong positive correlation was evident [8]. Although these scales are also not validated, they are frequently used to evaluate pain [12] and lameness in horses [13,14]. Correlation with such classical scales has also been used previously to validate pain scales in horses for measurement of visceral pain [3,4].

The discrepancy in variability between the evaluators and the standard evaluator for each item of the scale suggests poor criterion validity. However, another possible explanation would be the limited training of the evaluators, combined with the complexity and large number of items that comprise the scale. The blinded evaluators were chosen because of their experience in pain-related studies in numerous other species, including horses. Furthermore, the item-total correlation, which denotes the importance of each item, showed that most provided moderate correlation. Hence the very large number of items evaluated within the initial scale is likely to have reduced the overall criterion validity.

The variation in intra and inter-observer reliability for each item on the scale may suggest a low reliability of the proposed instrument under study. However, the process of pain scale validation does not occur in one step but is iterative, so after excluding items showing no specificity and relevance, the instrument should be re-evaluated using the same validity criteria [2,5,7]. The poor reproducibility for some items, such as “Response to approach” and “presence of the observer”, may be related to failure of observation, due to the difficulty in observing the videos, a fact that might be resolved when observations are performed *in situ*. Otherwise the presence of the observer may also modify the animals’ behaviour.

The scale items that gave the best relevance, specificity and total-item correlation results were retained in the scale after the refinement. However, despite the lack of relevance and low inter-observer reliability, the behaviour “kicking the abdomen” was retained in the scale as this is considered to be a classical abdominal pain related behaviour [12,15]. Although the inclusion of physiological parameters is questioned by some authors [3], these items are usually included in tools to assess acute pain in horses [2,5], as well as in other species [7] and provide a multidimensional character to the scale. Heart rate was retained after refinement as this was the only parameter that varied with time, it is easy to evaluate and has historical importance in the assessment of pain [12]. In view of the fact that heart rate increased above 25% of pre-operative values (TC) in animals undergoing surgery (GC and GCA) at T4 and T6, overall changes in heart rate above 25% were considered relevant as an indicator of post-operative pain and were therefore included.

As noted in a study that described the behaviours of horses undergoing arthroscopic surgery and laparotomy [16], horses without pain were more likely to position themselves at the front of the stable compared to other positions in the box. Behaviours such as “head position” and “response to auditory stimuli” were excluded due to their variability and since they might be unduly influenced by environmental stimuli.

Behaviours related to the interaction with the observer showed similar relevance and specificity to those reported when using an orthopaedic pain scale [2] and similar item-total correlation to animals undergoing laparotomy [5]. However this behaviour may also be influenced by the type of management with which the animal is familiar [17]. In our study, locomotion was also useful to detect pain after soft tissue surgery, as animals in pain tend to be reluctant to move, reflecting the findings of altered locomotion in horses after orthopaedic surgery [13,14]. However this contrasts with results from other studies in which increased locomotion was associated with pain [2,3], indicating that it is the change in locomotion that is a useful characteristic to evaluate during pain assessment in horses.

Although palpation of the surgical site showed low item-total correlation in this study, specificity ranged from moderate to good and this item was relevant. In a previous study, horses undergoing laparotomy showed a high incidence of avoidance responses [5]. In our study, the reaction response was probably related to the inflammation caused by surgical incision. However, it is common for horses not to tolerate palpation of the inguinal area. Furthermore, in those cases where this behaviour was evaluated on the video, there may have been misinterpretation. Although the two cameras were placed in diagonally opposite positions in the stable to try to avoid blind spots, it was difficult to observe the animal when it was positioned close to the wall directly beneath one of the cameras. Under some of these circumstances it was not possible to visualize the pelvic limbs during palpation of the groin.

This is the first study to identify the behaviour of lifting the pelvic limb as a pain-related behaviour in the horse, indicated by the relevance and moderate specificity and item-total correlation. This item was included in the scale after validation of content and before construct validation because it was a behaviour observed by the evaluator *in situ* during assessment of the GC group.

Since there is now a considerable body of work describing the development of tools for pain assessment, it was possible to evaluate the relevance, specificity and reliability of various pain behaviours previously described as relevant in horses. The low repeatability and reproducibility of some behaviours may indicate that their interpretation is influenced by the experience of the evaluator, and therefore they are imprecise. Although the reliability of the total score of the refined scale was not investigated, the sensitive and specific items of the behaviours and categories may be used to compose a refined scale for future validation, ideally under clinical conditions.

It should also be noted that during the initial part of the scale, the observer was not present in the box. I It is therefore difficult to accurately ascertain how much the evaluator's presence might interfere with the pain assessment. Consequently, whenever possible horses should be observed using a remote monitoring system. Although the time necessary for pain assessment has not been determined, after 700 hours of video analysis, we empirically suggest a time frame of 5 minutes would be sufficient for observation of pain-related relevant behaviours in the horse.

## Conclusions

In conclusion, this is, to our knowledge, the first study to refine and validate a pain scale for assessing acute, mild clinical pain in horses undergoing castration. The proposed new scale showed construct validity and responsiveness, and differentiated between horses with and without pain as reported previously in horses undergoing moderate and severe pain intensity, like orthopaedic and abdominal pain. Reliability of the initial items included in the scale was variable, suggesting the need for refinement of the scale; this led to selection of items that showed relevance, specificity, and item-total correlation. Refinement of the scale, and exclusion of items that did not meet the predefined validity requirements, provided a simple version for evaluation of postoperative pain after soft tissue surgery in horses that may be further tested under clinical and experimental conditions.

## Methods

The Institutional Animal Scientific Use Ethical Committee approved the study (protocol number 186/2009) and written informed consent was obtained from the owners before their horses were recruited to the study.

The acute pain scale was developed using previously published data [2,12] and by observing approximately 700 hours of videos before and after castration. Based on these data the behaviours of animals with or without pain were identified. Content validation review was based on evaluation of each item of the scale as relevant (1), irrelevant (−1) or not known (0) by three experienced equine veterinarians. The arithmetic mean was calculated for each item and those with values greater than or equal to 0.5 [8] were included in the scale. The scale composed 62 items with scores ranging from 0 to 3 and total score of 40 points (Table 1). Physiological parameters were also evaluated in addition to the items described in Table 1. Heart and respiratory rates and non-invasive systolic arterial blood pressure were evaluated according to the following criteria when compared to the initial (baseline) values: 0 – less than 10%; 1 – between 11 and 30%; 2 - between 31 and 50% and 3 – above 50% increase when compared to initial values. Intestinal sounds were evaluated as 0 – normal; 1 - decreased gut sounds; 2 – increase gut sounds or no gut sounds.

Construct validity was examined by contrast group analysis, comparing animals with or without pain. Twenty-four client owned adult horses confirmed as healthy following clinical and laboratory assessment were recruited and randomly (Excel®)^a^ allocated to one of four following groups: anaesthesia only (GA); pre-emptive analgesia and anaesthesia (GAA); anaesthesia, castration and postoperative analgesia (GC); or pre-emptive analgesia, anaesthesia and castration (GCA). The same experienced surgeon performed all castrations. All animals were housed in individual stables and allowed to acclimatize for at least 36 hours before any behavioural data were collected. Only well-handled horses were recruited to the study. The sample size was determined using an expected mean pain score difference between the groups of 4.0, with a standard deviation of 3.0, based on pilot studies, with a test power of 90% and 5% level of significance.

All horses were sedated with 0.5 mg/kg xylazine IM (Sedomin®)^b^, followed by induction of anaesthesia with 100 mg/kg of 10% guaiphenesin (Eter Gliceril Guaicol®)^c^ and 5.0 mg/kg of thiopentone IV (Thiopentax®)^d^. After orotracheal intubation, anaesthesia was maintained with isoflurane (Isoforine®)^d^ in oxygen. Ventilation was controlled (Mallard Medical®)^e^. Pre-emptive (GAA and GCA) or postoperative (GC) analgesia consisted of the administration of 0.2 mg/kg morphine (Dimorf®)^d^ IM, 10 mg/kg dipyrone (metamizol) (Finador®)^f^ IM and 1.1 mg/kg flunixin meglumine (Desflan®)^f^ IV. Local anaesthesia was provided with 10 ml of 2% lidocaine with adrenaline (Lidocaina®)^d^ injected into each spermatic cord before surgery in GCA. After recovery from anaesthesia the animals were transferred back to the observation stable, which was equipped with two video cameras (1.3 megapixels) placed in opposite corners at a height of 2 meters. The cameras provided colour images and were equipped with an infrared device to enable image capture under low light conditions. Video recording commenced immediately before anaesthesia and for 24 hours afterwards. Over this 24 hour period an investigator also assessed the animals directly by entering the stable and assessing pain in a standardised manner at the following time points: TC (before surgery and/or anaesthesia); T4 (four hours after anaesthetic recovery, before administration of systemic analgesia in the GC group); T6 (six hours after anaesthetic recovery) and T24 (24 hours after anaesthetic recovery). After the investigator entered the stable, the horse was approached and offered pelleted food in a small container. Pain assessments were then performed and after these were completed, the horse’s heart rate and intestinal motility [18] were assessed by auscultation, respiratory rate by observation of thoracic wall movements and systolic arterial blood pressure by the Doppler technique (Parks Medical 812®)^g^ with the probe and cuff positioned over the coccygeal artery. Following analysis of all of the video data, four 3 to 4 minute videos were generated for each animal at time points TC, T4, T6 and T24. These included footage recorded one hour before the presence of the investigator and during the time that the investigator was present in the stable undertaking the pain assessment. The duration of the video clips was sufficient for the included behaviours to be expressed by the horses.

The investigator (standard evaluator) and four experienced equine clinicians (evaluators) watched the videos on two different occasions at intervals of at least two weeks. The order of the videos was changed for the second assessment. The evaluators were blinded with respect both to treatment group (GA, GAA, GC, GCA) and to the assessment time point (TC-T24). The evaluators used the acute pain scale to assess pain in the horses, without any scores assigned to any item on the scale. The scores were subsequently included for statistical evaluation. The following instructions were given to the evaluators prior to watching each sequence of videos. 1) After watching each video clip answer the following questions according to your clinical experience fill in the numerical pain scale (1: without pain to 10: worst possible pain), followed by the simple descriptive scale (1: without pain to 4: severe pain) and then the visual analogue pain scale (0: without pain to 100 mm: worst possible pain); 2) Subsequently fill in the proposed pain scale choosing the descriptor level within each item that best represents what was observed; 3) If you are unsure at any time about what behaviours were shown in the video, the video may be replayed. Specific behaviours such as looking at the flank and lifting of hind limbs were considered after the behaviour had been observed once or several times.

### Statistical analysis

For content validity, only values equal to or greater than 0.5, obtained by the arithmetic mean of the scores given by the three evaluators for each item, were accepted and included in the pain scale [19].

The specificity of each item (defined by the ability of the test to correctly identify patients that were exhibiting pain behaviours calculated by the ratio between the true negatives and the sum of the true negatives and false positives), was evaluated by investigating if that particular behaviour was present or not at TC in all observations from all evaluators, in all animals from all groups. When a given behaviour was present in animals after surgery and likely feeling pain, but was not expressed or infrequently expressed in horses free of pain (TC), that behaviour was considered relevant to differentiate a horse with or without pain and therefore would be considered having high specificity. Specificity was classified as excellent (0–4.9%), good (5–14.9%), moderate (15–29.9%), or nonspecific (≥30%) [2].

The relevance of each item, i.e. the chance of observing a particular behaviour at T4 (when the most intense pain was expected) [2] was estimated by odds ratio using a logistic regression model for each item. An item was considered relevant when there was difference between GC *versus* GCA, GA and GAA, or when GC was different from GA and GAA, and irrelevant when there were no differences between GC and the other groups.

The total score of the refined scale was obtained by summing only the scores of the categories that showed items with relevance, specificity and item-total correlation. Categories that did not fulfil the above criteria were excluded from the sum of the total score of the refined scale (Table 4). Only the physiological variables showing changes over time were included in the sum of the total score of the refined scale.

The comparison of total scores between treatments was performed using the Kruskal-Wallis test and the difference between the scores over time in each group using the Friedman test. Construct validity was assessed by comparing the total score of the refined scale at the assessment time point where the animals were expected to have the most intense pain (T4 in GC and GCA) against the other time points (TC, T6, T24). Responsiveness was based on the percentage of change in pain score before and after administration of analgesia in groups GC [7,11], and by observing the difference between the groups in pain scores at time point T4 [2].

To investigate the criteria validity of each item, the Kappa coefficient was used to estimate the reliability of the score of the item between each evaluator and the standard evaluator, generating four kappa values; the values of each comparison were classified and grouped when reliability was similar [7,20]. The Pearson’s correlation coefficient was used to estimate the correlation between each variable (Table 2) against the total score of the proposed scale. In addition the correlation between the scores of the proposed scale and numerical, simple descriptive and VAS scale correlations was tested to investigate the convergent validity.

Intra- and inter-observer reliability for each item of the scale were assessed by use of the Kappa coefficient to compare differences in scores assigned on the first and second occasion that each video was watched by each evaluator, and by comparing scores assigned to the same video between different evaluators. Physiological data were investigated by repeated measures modelling evaluated for distribution and the gastrointestinal sounds were evaluated using the Wilcoxon test.

Statistical analyses were conducted with SAS version 9.3 [21] and differences were considered significant when p < 0.05.

### Endnotes

^a^Microsoft, EUA.

^b^Konig, Buenos Aires, Argentina.

^c^L.P.S. Agrofarma, Mogi Mirim, São Paulo, Brazil.

^d^Cristália, Lindóia, São Paulo, Brazil.

^e^Mallard Medical Skypark Drive Redding, CA, USA.

^f^Ourofino, Cravinhos, São Paulo, Brazil.

^g^Parks Medical Eletronics, Las Vegas, Nevada, USA.

## Abbreviations

GA: Group anaesthesia only
GAA: Group pre-emptive analgesia and anaesthesia
GC: Group anaesthesia, castration and post-operative analgesia
GCA: Group pre-emptive analgesia, anaesthesia and castration
TC: moment before surgery and/or anaesthesia
T4: moment four hours after anaesthetic recovery before administration of systemic analgesia in the GC group
T6: moment six hours after anaesthetic recovery
T24: moment 24 hours after anaesthetic recovery
IM: intramuscular
IV: intravenous
